# Determination of Albendazole and Metabolites in Silkworm *Bombyx mori* Hemolymph by Ultrafast Liquid Chromatography Tandem Triple Quadrupole Mass Spectrometry

**DOI:** 10.1371/journal.pone.0105637

**Published:** 2014-09-25

**Authors:** Li Li, Dong-Xu Xing, Qing-Rong Li, Yang Xiao, Ming-Qiang Ye, Qiong Yang

**Affiliations:** Sericulture & Agri-food Research Institute GAAS, Guangzhou, China; Uppsala University, Sweden

## Abstract

Albendazole is a broad-spectrum parasiticide with high effectiveness and low host toxicity. No method is currently available for measuring albendazole and its metabolites in silkworm hemolymph. This study describes a rapid, selective, sensitive, synchronous and reliable detection method for albendazole and its metabolites in silkworm hemolymph using ultrafast liquid chromatography tandem triple quadrupole mass spectrometry (UFLC-MS/MS). The method is liquid-liquid extraction followed by UFLC separation and quantification in an MS/MS system with positive electrospray ionization in multiple reaction monitoring mode. Precursor-to-product ion transitions were monitored at 266.100 to 234.100 for albendazole (ABZ), 282.200 to 208.100 for albendazole sulfoxide (ABZSO), 298.200 to 159.100 for albendazole sulfone (ABZSO_2_) and 240.200 to 133.100 for albendazole amino sulfone (ABZSO_2_-NH_2_). Calibration curves had good linearities with R^2^ of 0.9905–0.9972. Limits of quantitation (LOQs) were 1.32 ng/mL for ABZ, 16.67 ng/mL for ABZSO, 0.76 ng/mL for ABZSO_2_ and 5.94 ng/mL for ABZSO_2_-NH_2_. Recoveries were 93.12%–103.83% for ABZ, 66.51%–108.51% for ABZSO, 96.85%–105.6% for ABZSO_2_ and 96.46%–106.14% for ABZSO_2_-NH_2_, (RSDs <8%). Accuracy, precision and stability tests showed acceptable variation in quality control (QC) samples. This analytical method successfully determined albendazole and its metabolites in silkworm hemolymph in a pharmacokinetic study. The results of single-dose treatment suggested that the concentrations of ABZ, ABZSO and ABZSO_2_ increased and then fell, while ABZSO_2_-NH_2_ level was low without obvious change. Different trends were observed for multi-dose treatment, with concentrations of ABZSO and ABZSO_2_ rising over time.

## Introduction

Safety in silkworm egg production is critical for sericulture. Silkworm microsporidiosis is the primary reason for quarantine worldwide because of its germination transmission which can lead to devastating infectious disease [1]. Currently, drug therapy, particularly with benzimidazoles, is used to prevent and control silkworm microsporidiosis [2].

**Figure 1:**
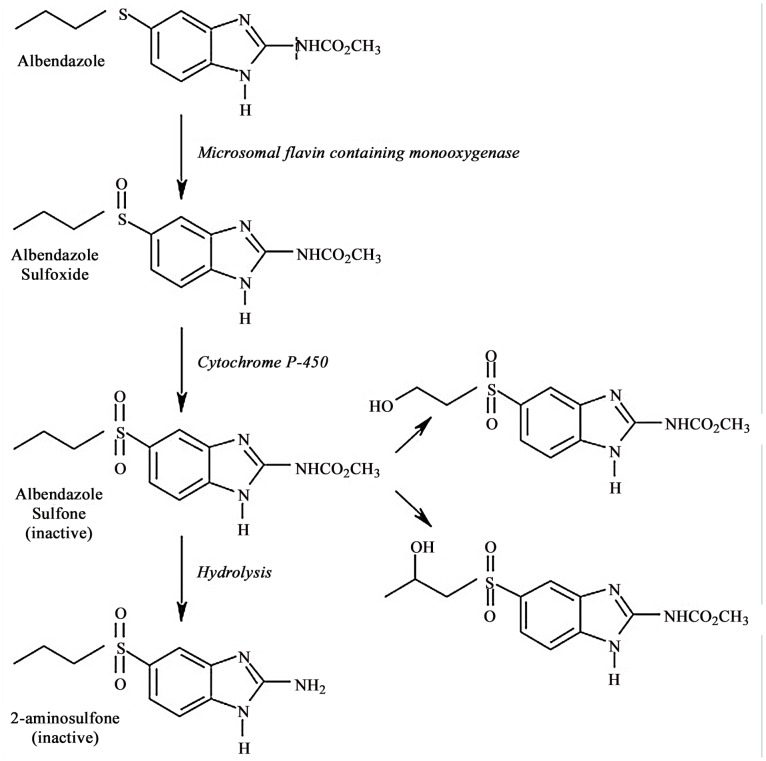
Chemical structure and main metabolic pathways of albendazole and metabolites in organisms (Gyurik et al. [8]).

Albendazole (ABZ) is a broad-spectrum benzimidazole parasiticide. ABZ is widely applied in the livestock and poultry industry and in aquaculture because of its high effectiveness and low host toxicity. Pharmacokinetic studies of ABZ have been conducted in mice, goats, cattle, sheep, rats, dogs, and humans [3]–[8]. The *in vivo* metabolism of ABZ is a pathway of ABZ to albendazole sulfoxide (ABZSO) to albendazole sulfone (ABZSO_2_) to albendazole amino sulfone (ABZSO_2_-NH_2_) [8]–[10] (Figure 1). ABZSO is the key active ingredient that inhibits parasites from absorbing glucose. This restricts the fumaric acid reductase system and prevents ATP production, leading to glycogen depletion, inviability and failure to reproduce. In the past decade, in research on silkworm microsporidiosis, our lab has screened reagents including ABZ, which has excellent therapeutic effects [11]. However, the treatment and mechanism of metabolism (pharmacology) and the bioavailability of ABZ in the silkworm are poorly understood. Few accurate methods for determining ABZ in silkworm hemolymph are available.

In the past 20 years, several methods for detecting benzimidazole in biological tissues or food have been developed including immunoassays, capillary electrophoresis, gas chromatography tandem spectrometry and high-performance liquid chromatography (HPLC). HPLC is often coupled with a refractive index detector, ultraviolet visible detector, variable wavelength detector, fluorescence detector or photodiode array detector. In the early 1980s, HPLC began being used for benzimidazole detection. The US Food Safety and Inspection Service recommends HPLC to measure residual ABZ content [12]. Comparing nonaqueous capillary electrophoresis and HPLC-UV as ABZ assays for human plasma, Prochazkova et al. [13] reported that the sensitivity and limit of detection (LOD) were similar for both techniques, but for measuring ABZO, nonaqueous capillary electrophoresis was a viable alternative to HPLC. Alvinerie and Galtier [14] simultaneously determined ABZ and its principal metabolites in sheep plasma using a normal-phase HPLC equipped with a UV detector, reporting LODs of them were 20–50 ng/mL, while ABZSO_2_-NH_2_ could not be detected. Lanchote et al. [15] used HPLC with a fluorescence detector to measure ABZSO and ABZSO_2_, and achieved lower limits of quantification (LOQs) of 5 ng/mL for ABZSO and 1 ng/mL for ABZSO_2_. Mottier et al. [16] successfully measured benzimidazole content inparasite material of sheep and cows using an HPLC technique similar to Kitzman [17]. Batzias and Delis [18] accurately quantitated ABZ and its metabolites in sheep plasma by reverse-phase liquid chromatography (RP-HPLC). However, recently, researchers have relied more on mass spectrometry (MS) detection than on conventional detection methods for confirmation of the presence of drugs. Liquid chromatography (LC) coupled with single MS [19], [20] or MS/MS [21], [22] allows quantification at trace levels and is a powerful technique for measurement of drugs and metabolites in biological fluids. Other analyzers such as time-of-flight MS or evaporative light-scattering MS can also be used [23], [24]. The first detection of benzimidazole residue in muscle, liver and egg using LC-MS-MS was reported by Balizs [25]. Bonato et al. [26] also accurately quantified ABZSO and ABZSO_2_ by LC-MS. According to Gonzalez-Hernandez et al. [22], LC-MS/MS was a quick, simple and reliable method for determining ABZSO and ABZSO_2_ in plasma and cerebrospinal fluid samples and therefore applicable for therapeutic monitoring.

The first commercial Ultra performance liquid chromatography (UPLC) system was marketed by Waters in 2004, followed by rapid-resolution liquid chromatography (RRLC) by Agilent and ultrafast liquid chromatography (UFLC) by Shimadzu. UPLC, RRLC and UFLC yield higher column efficiencies than traditional HPLC techniques [27], [28]. Nowadays, UPLC coupled with MS/MS is used by researchers because of advantages over HPLC or HPLC-MS/MS such as short injection cycle times, high sensitivity and resolution, an increased signal-to-noise ratio (S/N), reduced use of chemicals and production of waste, and a lower limit of quantification. Thus, UPLC-MS/MS is used for multiclass, multi-analyte, multi-residue detection and trace analysis [27]. A UPLC-MS/MS method is used to measure 23 veterinary nitroimidazoles, benzimidazoles and chloramphenicols in porcine tissues with higher sensitivity than previous methods [29]. Zhan et al. [21] reported that UPLC-MS/MS simultaneously detected 220 undesirable chemical residues, including four benzimidazoles, in infant formula. Previous studies suggested clear advantages of UPLC-MS/MS such as higher efficiency, higher sensitivity and faster detection than generic HPLC for veterinary drug analysis of biological materials. Effort and time can be reduce by application of UPLC-MS/MS in routine monitoring programs [21]. However, no UPLC, RRLC or UFLC methods are reported for rapid and simultaneous determination of albendazole and metabolites in silkworm hemolymph that are suitable for pharmacokinetic studies.

Silkworms are too small for easy and adequate sampling of hemolymph. When orally administered, ABZ, ABZSO, ABZSO_2_ and ABZSO_2_-NH_2_ (collectively ABZs) are present at low concentrations in silkworm plasma samples. In addition, endogenous substances such as inorganic salts, lipids, proteins and other metabolites make drug detection difficult in silkworm hemolymph. Given these silkworm characteristics, an appropriate method must be developed for the simultaneous and rapid determination of ABZs in silkworm hemolymph.

Therefore, we focused on establishing an accurate, simple, sensitive, rapid and suitable method for characterizing and quantifying ABZ and metabolites in silkworm hemolymph by ultrafast liquid chromatography tandem triple quadrupole MS (UFLC-MS/MS) coupled with electrospray ionization (ESI+) and multiple reaction monitoring (MRM). The method involved simple liquid/liquid extraction followed by UFLC separation and MRM detection. Variation of hemolymph-drug concentrations in silkworm at both single dose and multiple dose were also investigated. This study contributes to the theory and knowledge about test methods for ABZs in tissues from treated insects and provides technical information for pharmacokinetic studies.

## Materials and Methods

### Chemicals and reagents

All chemicals and reagents for ABZs extraction and UFLC-MS/MS analysis were of the highest available purity. Chromatographically pure acetonitrile was from Thermo Fisher Scientific Company and methanol was from Oceanpak Alexative Chemical, Ltd. For reference standards, ABZ (99.0%) and ABZSO_2_ (98.3%) were from Dr. Ehrenstorfer GmbH (Germany) and ABZSO (≥98%) and ABZSO_2_-NH_2_ (98%) were from Sigma-Aldrich (St. Louis, MO, USA) or Alfa Aesar (USA). Water was purified using a Milli-Q deionization unit (Millipore, Bedford, MA, USA).

### Feeding and sample preparation

The silkworm variety was Yanqi, from Mulberry & Silkworm Germplasm Resources, Guangdong Biological Germplasm Resource Bank (Guangdong province, China). A randomized block design was used. Treatments were of 600 silkworms and replicated three times. Silkworms were reared on fresh mulberry leaves under standard conditions (3 times a day) until tetramolter. ABZ-treated fresh mulberry leaves were generated by soaking leaves in 2000 mg/L ABZ suspension for a few seconds and air-drying before feeding. The first feeding with ABZ-treated-leaves was at the beginning of the fifth instar stage. Single-dose and multi-dose treatments were designed. For single treatments, 1.5 kg ABZ-treated leaves were provided for 4 h, followed by routine feeding. For multi-dose treatments, 27.0 kg ABZ-treated leaves were supplied in batches for 72 h covering the entire fifth instar stage. Silkworms fed on fresh mulberry leaves only were used as controls. In four hours, after first feeding with ABZ-treated leaves, haemolymph was sampling every 2 h. For each sample, the mixed hemolymph of 30 silkworms was harvested from a wounded cercus with scissors, immediately frozen in liquid nitrogen and stored at −20°C.

Hemolymph samples were homogenized (XW-80A, Kylin-bell Inc.) for one minute before extraction. Using Ruyck’s [30] method with modifications, samples were extracted with ethyl acetate-acetonitrile (1/5 v/v). An aliquot of 1 mL of sample homogenate was stirred in 5 mL of extracting solution for 15 min. After centrifugation at 10,000 × *g* for 5 min at 4°C (Universal 32 R, Hettich Inc., Germany), extraction of remaining residue was repeated with 2 mL of extracting solution. Combined supernatant was passed through a 0.22 µm polytetrafluoroethylene membrane (Tianjin Jinteng Experiment Equipment Co., Ltd., China) using a syringe filter, infused into an dedicated autosampler vial (Agilent, USA) with disposable sterilized syringes, sealed with parafilm (American National Can Company) and stored at 4°C as soon as possible for UFLC-MS/MS analysis within 24 h. For blank controls, hemolymph was sampled and prepared as above.

### Preparation of standard solutions

An external standard method was used for qualitative and quantitative determinations. For a standard stock solution of 20 mg/L, ABZ, ABZSO, ABZSO_2_ and ABZSO_2_-NH_2_ were weighed to 0.001 g using an electronic balance (FR224CN, Ohaus corporation, USA), dissolved together in 50 mL acetonitrile with stirring and stored at 4°C. Diluted mixed working standard solutions of 5, 62.5, 125, 250, 625 and 1 250 ng/mL were prepared in acetonitrile from the stock solution. Standard curves were made using the data from peak areas and corresponding concentration gradients of the four reference standards.

### Ultrafast liquid chromatography-tandem triple quadrupole mass spectrometry

The UFLC-MS/MS system was a GU-20A3 prominence degasser UFLC (SHIMADZU) and Applied Biosystems Sciex (AB Sciex, USA) API 4000 tandem triple quadrupole mass spectrometer. UFLC was equipped with automatic liquid chromatographic sampler (SIL-20AC), injector (SIL-20AXR), bi-pump (LC-20AD), column thermostat (CTO-20A) and degasser (LC-20AD). The chromatographic column, Agilent ZORBAX C18 (4.6 × 100 mm, 3.5 µm) was kept at the column temperature of 40°C. Isocratic elution used a mobile phase of acetonitrile: 0.005 mol/L formic acid (v/v) 85%/15%. The sample injection volume was 10 µL. The flow rate was 0.2 mL/min. An API 4000 tandem triple quadrupole MS with ESI interface was operated in ESI+ mode using parameters: atomizer, 7.00; curtain gas, 25.00; collision gas, 5.00; ionspray voltage, 5500.00 V; ionization temperature, 550.00°C; injection voltage, 10.00 V; and collision cell voltage, 10.00 V. Nitrogen was used in all cases. Signal acquisition and analyte quantification were carried out by MRM acquiring the precursor-product ion pairs of the transitions. In ESI(+)[M+H]+ MRM model, mass spectrum parameters and m/z of ion pairs are listed in Table 1.

**Table 1:** Mass spectrum parameters of albendazole and metabolites.

Components	Qualitative ionpair *m/z*	Quantitative ionpair *m/z*	Declustering potential(DP, V)	Collision energy(CE, eV)	Focus voltage(FP, V)
ABZ	266.100→191.100	266.100→234.100	131.00	45	190
	266.100→234.100			28	
ABZSO	282.200→191.100	282.200→208.100	128.00	54	170
	282.200→208.100			34	
ABZSO_2_	298.200→224.100	298.200→159.100	142.00	38	185
	298.200→159.100			52	230
ABZSO_2_-NH_2_	240.200→198.00	240.200→133.100	180.00	43	190
	240.200→133.100			27	

### Method validation

The analytical method was validated following the typical-fundamental criteria of bioanalytical methods described as Buick et al. [31]. For assessing the reliability and overall performance of the UFLC-MS/MS method, specificity, LOQ, LOD, accuracy, precision, linearity, recovery, stability, and applications of the analytical system were evaluated. During the procedure, Bernoulli trial principles were maintained.

Specificity was assessed to determine whether analytes were clearly identified without interference from other compounds in the sample or not. A zero and a blank plasma sample were prepared for each analytical run to determine interference. One hundred and twenty five ng/mLof reference standard in the mobile phase were also prepared and analyzed.

Sensitivities for analytes were evaluated by LOD and LOQ values, defined as the lowest concentration that could be reliably and reproducibly detected and measured in at least three replicates. Under the chromatographic conditions of this method, LODs and LOQs were estimated as concentrations of analytes that yielded a S/N of 3 for LOD and 10 for LOQ. With a 10 µL injection volume, the chromatographic peak S/N of reconstructed ions of qualitative ion pairs in quality control (QC) plasma samples (62.5 ng/mL of each analyte) were used as calibrators to calculate LOD and LOQ.

Analyses of low (125 ng/mL), middle (625 ng/mL) and high (1250 ng/mL) drug concentration of QC samples were prepared separately and measured in the same manner within one day to verify the precision and accuracy of intraday (the same day) and interday (three consecutive days). Accuracy was expressed by bias calculated as: accuracy (bias, %) = [(A−Ā)/A]×100 where A was individual concentration and Ā was mean concentration. Precision was expressed by relative standard deviation (RSD) calculated as precision (RSD, %) = (standard deviation [SD]/Ā]×100 [24], [32].

Matrix-fortified calibration curves for quantification were established by linear regression analysis of peak area (as ordinate) versus concentration (as abscissa). Blank samples were used to calibrate standard levels. Sample concentrations were derived from the calibration curve. Abscissa points were 20 mg/L of mixed standard stock solution diluted to standard working solutions of 5, 62.5, 125, 250, 625 and 1250 ng/mL. ABZs concentrations in plasma samples were calculated with regression equations.

Recoveries were calculated at low, medium and concentration levels in QC samples with added 6.25, 31.25 and 62.5 µL of mixed standard stock solution. Fortified samples were treated using the extraction procedure above. Method repeatability was expressed as RSD of the replicated measurement (n = 6).

To investigate freeze-thaw stability, the QC samples of 125, 625 and 1250 ng/mL mixed stand stock solution for freeze-thaw stability tests were put in 3 cycles of 12 h at −20°C and 12 h at 4°C. Aliquots of the same sample were stored at 4°C for 3 days. Daily sampling and measurements were conducted. Results were expressed as RSDs of peak areas in the stability test.

### Application of the analytical system in a pharmacokinetics study

To determine practical applications of this UFLC-MS/MS method to quantify ion pairs of ABZs, silkworm hemolymph from single-dose treatment and multi-dose treatment was sampled at 2 h intervals until 12 h and measured by the method established.

### Statistic analysis

Signal analysis, and automatic integration and generation of signal charts used Analyst software (version 1.5.2; AB SCIEX, USA) matched for the UFLC-MS/MS equipment. Calculation of target components, RSDs and bias, and linear-regression analysis used Microsoft Office Excel 2003 or SPASS (Version 17.0) software.

## Results and Discussion

### Sample preparation

The high content of protein and fat in silkworm hemolymph influences drug extraction. Ethyl acetate is recommended by the Food Safety and Inspection Service, United States Department of Agriculture, to extract ABZ [31]. Acetonitrile affects protein precipitation and improves recovery [33]. Aguilera-Luiz et al. [23] successfully extracted milk samples with acetonitrile without sample clean-up to measure veterinary drugs using UPLC-MS. In this study, a mixed organic ethyl acetate-acetonitrile solvent was used for extraction of ABZs as described by Ruyck [30]. Liquid-liquid extraction might have led to interference from impurities and poor repeatability [34]. Thus in this study, a chromatographically pure solution was used for extraction, but olid phase extraction columns (SPE) were not used to save costs and simplify pretreatment. For full extraction, two optimal extraction times were chosen.

### Chromatographic and tandem mass spectrometric conditions

ABZ and metabolites are relatively stable in formic acid. To improve sensitivity, adding acid to improve ionization efficiency has been suggested [23], [24], [35]. We found that 15% formic acid in the mobile phase gave a higher signal response and better peak sensitivity. The acetonitrile-formic acid system provided good separation, an appropriate retention time and low background noise, and was selected as the mobile phase for detection. Previous research on ABZ determination suggested using advanced UPLC columns and a low flow rate (≤0.20 mL/min) for HPLC analysis, because using UPLC columns for bioanalytical methods results in higher peaks than obtained using HPLC columns while retaining high analysis quality [36]. We adopted the same flow rate of 0.2 mL/min for our method. To avoid co-elution of ABZ and metabolites, we developed a sensitive and accurate UFLC-MS/MS method for detecting ABZs in silkworm hemolymph.

**Figure 2:**
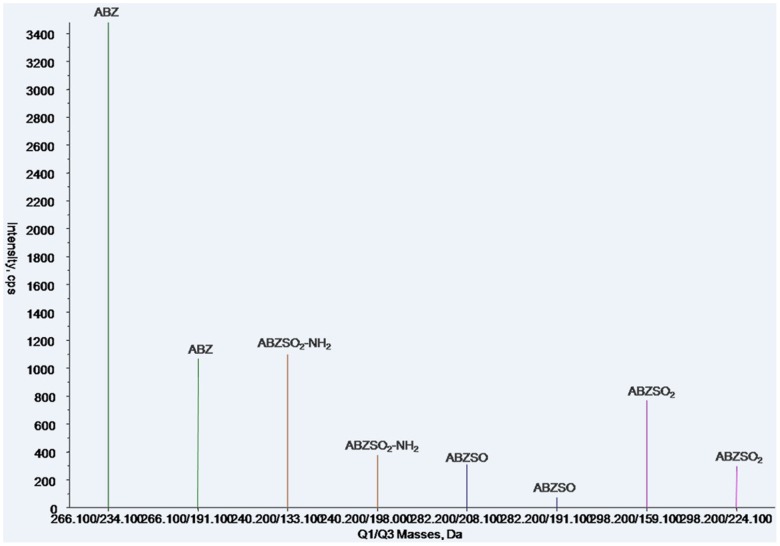
Total ion mass spectra of albendazole and metabolites. ABZ, albendazole; ABZSO, albendazole sulfoxide; ABZSO_2_, albendazole sulfone; ABZSO_2_-NH_2_, albendazole amino sulfone. Adjacent columns with the same color are two fragment ions from the same target analyte.

**Figure 3:**
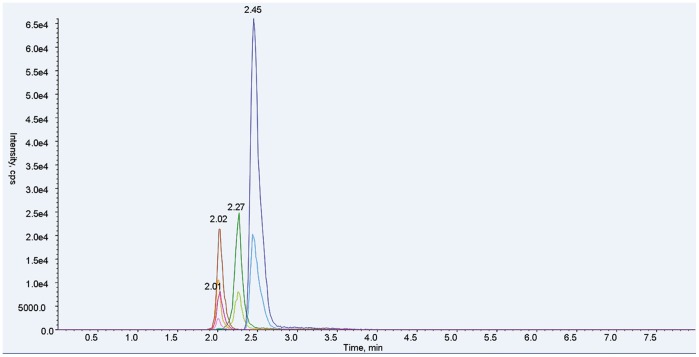
Representative UFLC-MS/MS MRM chromatogram of albendazole and metabolites. Peaks with the same retention time and approximate color are fragment ions from the same target analyte. Retention times: ABZ, blue, 2.45 min; ABZSO, red, 2.01 min; ABZSO_2,_ orange, 2.02 min; ABZSO_2_-NH_2_, green, 2.27 min.

**Figure 4:**
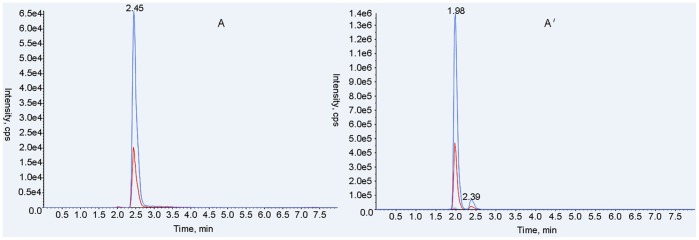
Representative UFLC-MS/MS MRM chromatograms of albendazole (m/z 266.100>234.100) in standard substance (A) and the selected sample (A′). Signals from different fragment ions are in blue and red.

**Figure 5:**
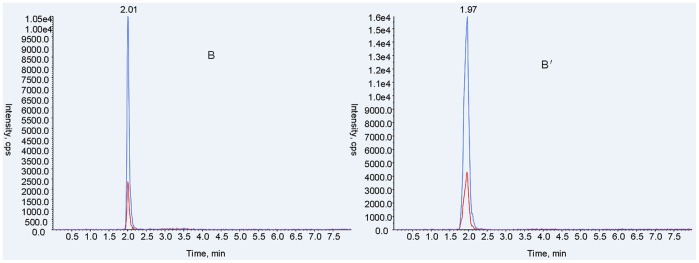
Representative UFLC-MS/MS MRM chromatograms of albendazole sulfoxide (m/z 282.200>208.100) in standard substance (B) and the selected sample (B′). Signals of different fragment ions are in blue and red.

**Figure 6:**
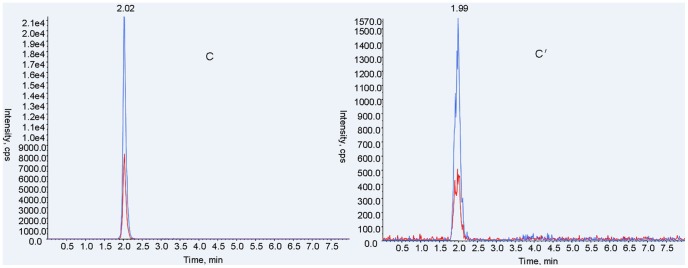
Representative UFLC-MS/MS MRM chromatograms of albendazole sulfone (m/z 298.200>159.100) in standard substance (C) and the selected sample (C′). Signals of different fragment ions are in blue and red.

**Figure 7:**
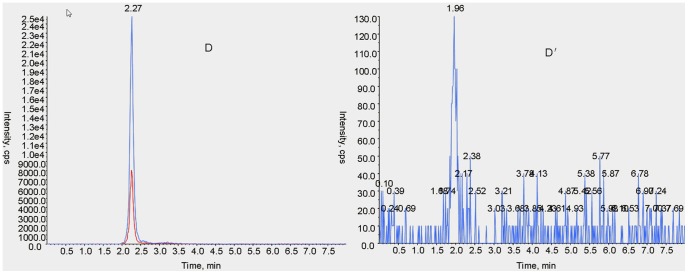
Representative UFLC-MS/MS MRM chromatograms of albendazole amino sulfone (m/z 240.200>133.100) in standard substance (D) and the selected sample (D′). Signals of different fragment ions are in blue and red.

In this method, both qualitative and quantitative analysis of ABZ and metabolites used an ESI+, MRM system. MS/MS parameters were manually adjusted for the highest response for each precursor and product ion combination, according to Shaw et al. with modifications [24]. Characterization of ion pairs of ABZ, ABZSO, ABZSO_2_ and ABZSO_2_-NH_2_ are in Table 1 with precursor ion (MS 1) and specific fragment (MS 2). The total ion mass spectra for ABZs was showed in Figure 2. A main advantage of UPLC over conventional LC methods is speed, allowing the determination of selected compounds in less than 10 min [23]. Hence, the improvement of the chromatographic system focused more on short retention times instead of chromatographic separation [24]. Under the selected chromatographic conditions, retention times for ABZs were 2.45, 2.01, 2.02 and 2.27 min. All analytes were separated within a 3-minute run and showed an integral MRM-chromatogram of mixed standard solution (Figure 3). ABZs of samples were consistent with the results of mixed standards for the approximate retention time, presenting good, symmetrical peak shapes and minor interferences from impurity peaks. Representative chromatograms of standard substances and hemolymph samples are in Figures 4–7. The relative ion abundance of qualitative ion pairs conformed with European Union principles for MS qualitative assays (Table 2). Therefore, this chromatographic conditions achieved good resolution and appropriate ionization for determining ABZs in silkworm hemolymph samples, even in the presence of endogenous metabolites and co-elutions of ABZs.

**Table 2:** Maximum permissible deviation of relative ion abundance.

Relative ion aboundance	>50%	>20%–50%	>10%–20%	≤10%
Maximum permissible deviation	±20%	±25%	±30%	±50%

### Specificity

Specificity was determined by analyzing mobile phase, blank hemolymph sample and spiked sample. For all of the four target analytes, no significant interference affecting quantification by retention times was observed using the optimized pretreatment processes.

### Limits of detections and limits of quantifications

LOD and LOQ were defined as the lowest concentration of analytes for which S/N was greater than or equal to 3 for LOD and 10 for LOQ. LODs and LOQs for ABZ of this study (Table 3) were lower than that in previous studies which used HPLC or HPLC-MS on human plasma [37], sheep plasma [14], [18] and milk samples [30]. In contrast, the LOQ for ABZSO was higher than that reported by Bonato [26], in which an optimized method was used for ABZSO and ABZSO_2_ determination. In general, the trends of our data were similar to previous studies, with higher LOD and LOQ of ABZSO than that of other target compounds. A possible explanation is that ABZSO is the main metabolic intermediate and active constituent and is not easily detected because of interference from its substrate and metabolites or other components. Nevertheless, the values we obtained were low enough to use this method in silkworm pharmacokinetic studies. Our results suggested high sensitivity of this UFLC-MS/MS method for measurement of ABZs in silkworm hemolymph.

**Table 3:** Recovery (%), repeatability (RSD%), LODs and LOQs of albendazole and metabolites in QC samples (n = 6).

Compounds	Concentration (ng/mL)	MRM 1 (quantification)	MRM 2 (confirmation)	Recoveries (%)	RSDs (%)	LODs (ng/mL)	LOQs (ng/mL)
ABZ	125	266.100234.100	266.100191.100	99.11	1.35	0.39	1.32
	625			103.83	2.74		
	1250			93.12	1.85		
ABZSO	125	282.200208.100	282.200191.100	66.51	18.69	5.00	16.67
	625			108.51	4.40		
	1250			96.23	2.01		
ABZSO_2_	125	298.200159.100	298.200224.100	97.96	7.63	0.23	0.76
	625			105.96	5.01		
	1250			96.85	4.18		
ABZSO_2_-NH_2_	125	240.200133.100	240.200198.000	99.64	3.99	1.78	5.94
	625			106.14	1.71		
	1250			96.46	4.97		

Note: definitively identified metabolites using chemical standards analyzed on the same system with the same analytical method.

### Accuracy and precision

The precision and accuracy of the method were assessed by replicate analyses of spiked samples (Table 4). Measurements of intraday and interday precision had RSDs from 0.2% to 7.6%. Bias of accuracy was from −9.9 to 7.8%. These results indicated good accuracy for the determination of ABZ and metabolites on the same or different days.

**Table 4:** Intraday and interday precision and accuracy for quantification of albendazole and metabolites from silkworm samples (n = 6).

Reference concentration(ng/mL)	Intra-day			Inter-day		
	Measured concentration(ng/mL)	Precision(RSD, %)	Accuracy(bia, %)	Measured concentration(ng/mL)	Precision(RSD, %)	Accuracy(bia, %)
ABZ						
125	125.4±3.6	2.9	0.3	125.2±0.4	0.4	0.1
625	648.9±17.8	2.7	3.7	663.5±17.2	2.6	5.8
1250	1207.4±32.8	2.7	−3.5	1210.1±7.1	0.6	−3.3
ABZSO						
125	113.7±7.2	6.3	−9.9	115.7±1.9	1.6	−8.0
625	678.2±29.8	4.4	7.8	666.5±16.8	2.5	6.2
1250	1202.8±24.1	2.0	−3.9	1193.5±17.4	1.5	−4.7
ABZSO_2_						
125	122.5±9.3	7.6	−2.1	122.6±2.3	1.9	−2.0
625	662.3±33.2	5.0	5.6	654.7±6.7	1.0	4.5
1250	1210.7±50.7	4.2	−3.2	1211.2±13.8	1.1	−3.2
ABZSO_2_-NH_2_						
125	124.6±5.0	4.0	−0.4	125.1±0.4	0.3	0.0
625	663.4±11.4	1.7	5.8	664.3±1.1	0.2	5.9
1250	1205.7±59.9	5.0	−3.7	1200.3±7.6	0.6	−4.1

Note: concentrations are expressed as mean ± standard deviation (STDEV).

### Linearity

Linear-regression analysis used peak areas and corresponding concentrations of diluted mixed working solutions at 5, 62.5, 125, 250, 625 or 1250 ng/mL. Correlation coefficients, residual plots and regression statistics were from regression analyses [38]. Good linearity was observed for the four target compounds. The mean values of regression equations for silkworm hemolymph were peak area (PAR) = 3.7242 × 10^6^C +3.4938 × 10^4^, R^2^ = 0.9927 for ABZ; PAR = 6.4613 × 10^5^C − 4.8495 × 10^3^, R^2^ = 0.9905 for ABZSO; PAR = 1.0269 × 10^6^C +89.1365, R^2^ = 0.9972 for ABZSO_2_; and PAR = 1.5396 × 10^6^C − 8.4586 × 10^3^, R^2^ = 0.9966 for ABZSO_2_-NH_2_. Regression analysis of the data showed a linear response between drug concentration and peak area in the examined range, with all coefficients of correlation greater than 0.99. The above four equations were used to calculate the contents of ABZs by peak areas of samples.

### Recovery

QC samples at low, medium and high concentrations of standard mixture compounds were prepared and extracted. The minimum recoveries of 93.12% for ABZ, 66.51% for ABZSO, 96.85% for ABZSO_2_ and 96.46% for ABZSO_2_-NH_2_ (Table 3), slightly exceeded those in human serum reported by Mirfazaelian et al. [39]. These results indicated good reproducibility of the detection method.

### Freeze-thaw stability

RSDs of peak areas for stability tests indicated that ABZ, ABZSO, ABZSO_2_ and ABZSO_2_-NH_2_ had sufficient stability in 12 h freezing at −20°C and 12 h at 4°C for 3 cycles or after storing at 4°C for 3 days (Table 5).

**Table 5:** RSDs (%) of sample peak areas in stability test (n = 6).

Concentration (ng/mL)	RSDs (%)							
	ABZ		ABZSO		ABZSO_2_		ABZSO_2_-NH_2_	
	Freeze-thaw cycle	Constant 4°C	Freeze-thawcycle	Constant 4°C	Freeze-thawcycle	Constant 4°C	Freeze-thawcycle	Constant 4°C
125	1.36	1.02	1.92	3.88	3.82	4.36	3.77	4.86
625	2.25	1.95	0.98	2.41	0.92	4.09	5.46	5.47
1 250	1.12	2.66	1.33	1.20	7.30	4.35	3.11	4.39

Note: definitively identified metabolites using chemical standards analyzed on the same system with the same analytical method.

### Application of the analytical system in a pharmacokinetics study

**Figure 8:**
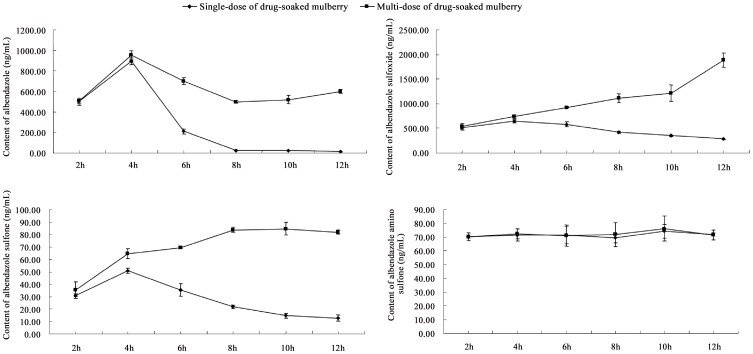
Albendazole, albendazole sulfoxide, albendazole sulfone and albendazole amino sulfone in silkworm hemolymph after feeding with albendazole-treated mulberry leaves.

To study the practical application of the UFLC-MS/MS method of quantifying ion pairs of ABZs, silkworm hemolymph was sampled at different time points. Single-dose treatment resulted in increasing and then decreasing of plasma drug concentrations of ABZ, ABZSO and ABZSO_2_, which was consistent with the reports using human plasma [17], [37] but not with the reports using mouse plasma [6]. No obvious change was observed in ABZSO_2_-NH_2_ concentration, which remained low. Multi-dose treatment resulted in different trends for ABZSO and ABZSO_2_. ABZSO showed a significant increase in concentration and ABZSO_2_ slightly rose over 10 h (Figure 8).

## Conclusion

UPLC-MS/MS technology has had a large impact on veterinary drug residue analysis and vermifuge and related compounds. Since UPLC-MS/MS became commercially available 10 years ago, injection cycle times have decreased, sensitivity has improved, resolution has increased and the number of compounds in multiresidue samples is rising [27].

Silkworms are small with limited drug intake, posing challenges to hemolymph sampling and creating difficulties in assays. In this study, an UFLC-MS/MS assay was developed and used to monitor ABZs in samples of medicated silkworm hemolymph. The method involved a simple liquid/liquid extraction followed by UFLC separation and a triple quadrupole system with MRM detection. In this method, acetonitrile eliminated interference from high-abundance proteins in silkworm hemolymph. The ‘extra column effect’ was reduced by using a small column tube and flow cells with small UFLC volumes, achieving 10 µl ultrafast injection and chromatographic separation for all analytes within a few minutes. The combination of UFLC and MS-MRM achieved high separation efficiency with rapid qualitative and quantitative detection of ABZ and its metabolites in silkworm hemolymph.

Based on this method, drugs in silkworm hemolymph harvested at different time points were measured and preliminary variation in drug concentration over time was described. A complete pharmacokinetic study of ABZ requires additional other method improvements providing more information on the ABZ metabolic pathway and on ABZ formulations for sericulture. Two specific points would improve this study: It might be technically feasible to combine SPE with UFLC-MS methods online to improve sample purity. Use of C18 SPE for cleaning drug-containing samples before UFLC injection might remove impurities and interference [17]. Furthermore, quantitation of ABZ, ABZSO, ABZSO_2_ and the two enantiomers of ABZSO [6], the main active ingredient, might be preferable.

Although our focus was the use of UFLC-MS/MS for determination of ABZs, we note that high resolution mass spectrometry is likely be a powerful alternative to MS/MS detection [27] for potential improvement of detection of ABZs.
